# Cytokines in equine platelet lysate and related blood products

**DOI:** 10.3389/fvets.2023.1117829

**Published:** 2023-03-09

**Authors:** Julia Moellerberndt, Alina Hagen, Sabine Niebert, Kathrin Büttner, Janina Burk

**Affiliations:** ^1^Equine Clinic (Surgery, Orthopedics), Justus-Liebig-University Giessen, Giessen, Germany; ^2^Unit for Biomathematics and Data Processing, Faculty of Veterinary Medicine, Justus-Liebig-University Giessen, Giessen, Germany

**Keywords:** platelet lysate, equine, IFN-γ, TNF-α, IL-1β, IL-4, IL-6, IL-10

## Abstract

In equine medicine, the use of regenerative therapeutics has gained growing attention, but is still a new and complex field with room for improvement. Platelet lysate (PL) can be used as therapeutic agent but is also a promising supplement for the culture of multipotent mesenchymal stromal cells. To enable a targeted use of PL both in clinic and laboratory, it is crucial to learn more details on its effective ingredients. While so far, mainly growth factor components have been analyzed in platelet-based products such as PL, the current study focuses on the content of cytokines in serum, plasma, platelet concentrate and PL. Blood was harvested from 20 clinically healthy horses and subjected to blood count and chemistry analysis, as well as to further processing to PL. Plasma and platelet concentrate were produced by a buffy-coat-based method and PL was produced from the concentrate by freeze-thawing. Samples from each horse were analyzed regarding interleukin (IL)-1β, −4, −6 and −10, interferon-γ and tumor necrosis factor-α concentrations using sandwich ELISAs. Cytokine concentrations in serum, plasma, concentrate and PL were similar and correlated significantly. However, there was a large inter-individual variability in cytokine concentrations between the different donor horses. The samples from some donor animals had overall very high cytokine concentrations, while samples from other donors had no measurable cytokine ingredient. This pattern was observed for all cytokines. There was a noticeable link between high cytokine concentrations in the blood products and abnormal findings in blood chemistry. Cytokine concentrations in samples from horses with abnormal findings were significantly higher than in samples from the remaining horses. The interindividual differences in cytokine concentrations could be highly relevant when using PL for therapy and cell culture, as the mode of action of the PL is likely changed depending on the presence of pro- and anti-inflammatory cytokines. Blood chemistry might be useful to predict cytokine concentrations in blood products.

## 1. Introduction

Platelets play a crucial role in hemostasis and contain various cytokines, chemokines and growth factors, which are released after activation (1). These messenger substances cause a further release of soluble mediators, initiating signaling cascades e.g., for inflammation regulation, angiogenesis or tissue regeneration (2, 3). Besides their biological functions in the body, platelets are being harvested for biomedical purposes, which includes their use in regenerative medicine in the form of platelet concentrate or platelet lysate (PL).

Platelet concentrate is mainly being used for therapeutic purposes (4, 5). However, when further processed to PL by using freeze-thaw cycles to disrupt the platelets (6, 7), their contents are released (6, 8–10), and shelf life and storability of the blood product are improved (11). Therefore, PL can not only be used as a direct therapeutic agent for various indications such as tendon disorders, corneal defects or to support wound healing in horses and humans (12–14), but also as a cell culture supplement (15) which can replace the ethically critical fetal bovine serum (16, 17).

PL as cell culture supplement is particularly useful for the culture of multipotent mesenchymal stromal cells (MSC), which are being explored as therapeutic agent in their own right. MSC culture with addition of PL not only preserves their proliferation and basic properties, such as plastic adherence, expression of specific surface antigens, and trilineage differentiation potential (18), but also has a positive impact on their efficacy. Overall, PL supports the unfolding of diverse biological activities of MSC so that they can exert proregenerative, anti-inflammatory, antifibrotic, and immunomodulatory effects (10). Accordingly, we have previously demonstrated that equine PL, produced by a scalable buffy-coat method, supports equine MSC proliferation and increases their pro-angiogenic potency (19). However, it has not been fully elucidated which PL components contribute to these beneficial effects.

To date, PL has been analyzed predominantly regarding its growth factor contents, while the possible presence of inflammation mediators in platelet-based blood products has been widely disregarded so far. This is surprising, considering the discussion on platelet rich plasma leukocyte contents and considering that the mode of action of other blood products such as conditioned serum relies on (anti-)inflammatory mediators. Knowledge on the cytokine ingredients of platelet-based blood products would be tremendously helpful for their targeted therapeutic use (20, 21). Moreover, the presence of cytokines in PL could play a crucial role for MSC culture, as it could affect their mode of action, for example by inflammatory licensing (22).

To close this gap of knowledge, in this study, we aimed to characterize and quantify different cytokines in equine PL. We show that the cytokine levels vary heavily between individual horses and that abnormal findings in blood chemistry might be indicative of high cytokine levels.

## 2. Materials and methods

### 2.1. Donor health status

Blood was collected from 20 horses (4–15 years; 14 mares, 5 geldings, 1 stallion) as approved by the responsible authority (regional council Giessen, A14/2019). Beforehand, the donor health status was evaluated by general clinical examination and only animals that were free of abnormal clinical findings were included. In addition to the whole blood intended for platelet lysate production, blood was drawn into EDTA and lithium heparin as well as serum blood collection tubes and analyzed in the laboratory. This included complete blood counts and blood chemistry analysis using an ADVIA 2120i with Multispecies software MS 5.9 (Siemens Healthcare GmbH, Erlangen, Germany). With the latter, electrolytes, urea, creatinine, total protein, albumin, globulins, bilirubin, alkaline phosphatase, glutamate dehydrogenase, γ-glutamyltransferase, aspartate aminotransferase, creatine kinase and lactate dehydrogenase were measured.

### 2.2. Blood processing

Whole blood was collected and processed to produce PL as described in detail previously (6). Briefly, using a buffy-coat based approach, platelet concentrate was produced from whole blood collected in CPD-loaded blood collection bags by centrifugation and blood separation steps. The PL was then produced from this concentrate by freeze-thaw cycles, centrifugation and filtration. Samples from plasma, platelet concentrate and PL were frozen and stored at −80°C for cytokine analyses. Additionally, serum samples were handled accordingly.

### 2.3. Cytokine measurements

Cytokine concentrations were analyzed in serum, plasma, platelet concentrate and PL from each horse using Equine DuoSet ELISA kits (R&D Systems, Minneapolis, MN, USA) for IFN-γ, TNF-α, IL-1β, IL-4, IL-6 and IL-10. The plates were read in an Infinite F50 plate reader and raw data were processed with the corresponding Magellan software (Tecan Ltd., Maennedorf, Switzerland).

First, different serum dilutions were tested for all horses in order to establish the experimental setup. The results showed considerable inter-individual differences in cytokine concentrations demonstrating the need to adjust the dilution factor for each donor. To estimate the possible influence of serum matrix effects in different serum dilutions, spiking experiments were performed. For this purpose, serum from horses with no detectable cytokine content was pooled and a dilution series (1:1, 1:10, 1:100, 1:1,000 in RD buffer) was prepared. These diluted serum samples were then spiked with cytokine standard, corresponding to the second highest concentration of the assay standard curve for each respective cytokine. The spiked samples were subjected to the remaining assay procedure according to the manufacturer's instructions.

**Figure 1:**
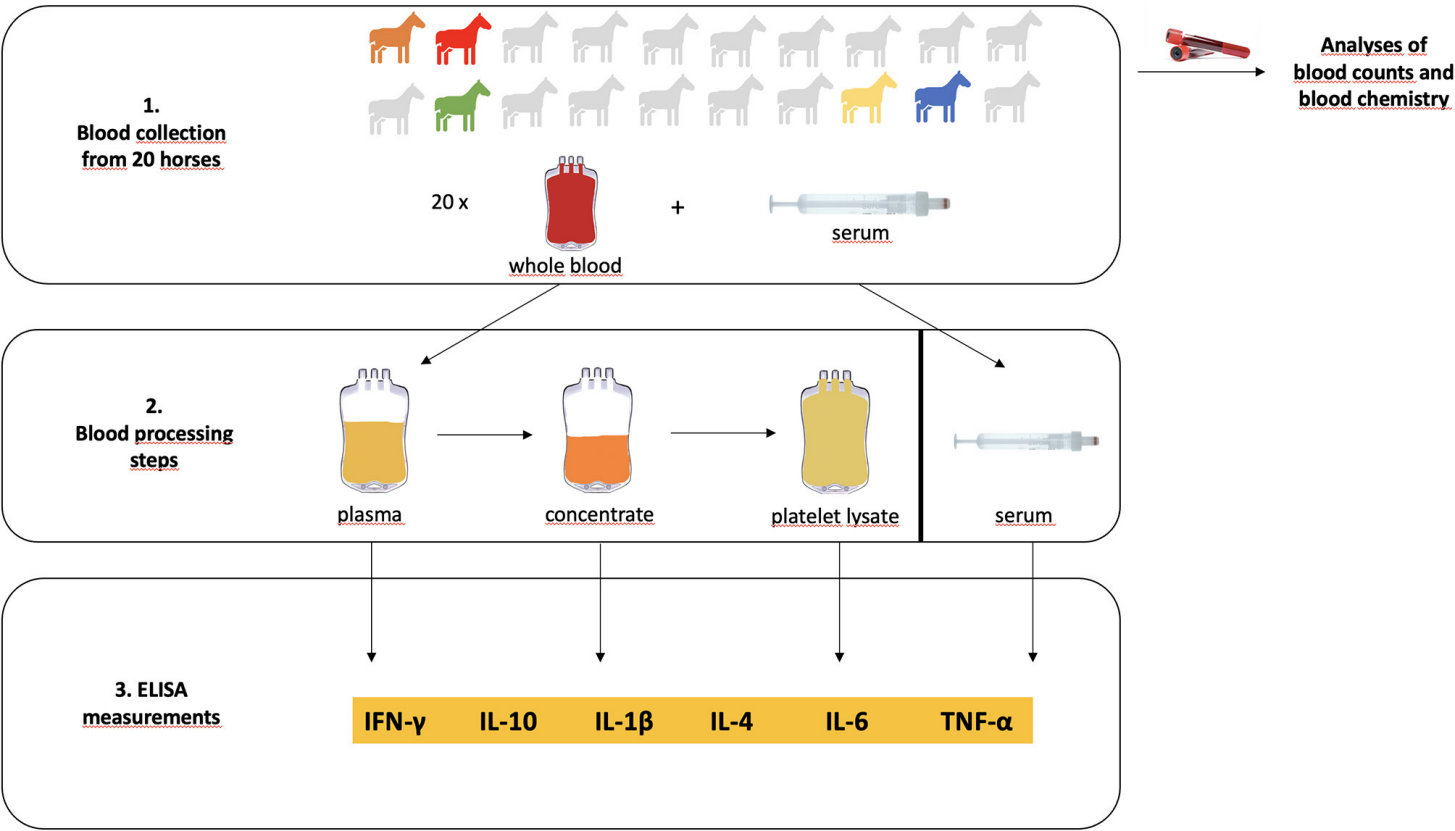
Overview of the study design. Blood was collected from 20 clinically healthy horses, of which 5 turned out to have mildly to moderately abnormal findings in blood chemistry (designated by the colored icons). Whole blood was processed to platelet lysate. Serum, plasma, concentrate and platelet lysate were then subjected to ELISA measurements of their cytokine contents.

The cytokine measurements in all blood products were performed for all cytokines using the ELISA assays according to the manufacturer's instructions. Blood product dilutions were adjusted individually, aiming to use the highest dilutions yielding results within the standard curve ranges in each case. Nevertheless, some samples had to be measured with no or low dilution. Samples with no detectable cytokine content in undiluted samples were assigned the value 0 for graphical presentation. The overall study design is shown in Figure 1.

### 2.4. Statistical analysis

Statistical analyses and graphical presentation of data were performed using IBM SPSS Statistics 26. Based on the results of the spiking experiment, we considered that the cytokine concentrations measured in blood product samples with low to medium cytokine content might be underestimated to some extent, as these samples could not be diluted enough to overcome the anticipated matrix effects. Therefore, for the statistical comparisons, cytokine concentrations were transformed into categories to circumvent calculations with inaccurate values. Category 1 corresponds to no detectable cytokine content, category 2 corresponds to the clusters with low to medium cytokine content, and category 3 reflects the clusters with high cytokine content. Non-parametric tests for paired samples were run to compare the different blood products, and non-parametric tests for unpaired samples were run to compare the donors with physiological blood chemistry and those with abnormal blood chemistry findings. In addition, possible correlations were evaluated according to Spearman.

## 3. Results

### 3.1. Donor health status

With respect to the general clinical findings, all horses were considered as healthy. Measurements of blood cells were within the reference range, except for very mild changes in two animals (18 and 19). Blood chemistry measurements were within the reference range in 11 horses, showed very mild changes in four horses (4, 5, 13 and 17) and mild to moderate elevations of either alkaline phosphatase, creatinine, lactate dehydrogenase in five horses (horses 1 and 18, horse 2, horses 12 and 19, respectively) (Table 1). The latter were considered as abnormal in the further analyses.

**Table 1:** Findings in blood counts and blood chemistry.

**Donor**	**Elevated parameter**	**Value**	**Reference values**
1^*^	Alkaline phosphatase	296 U/L	<260 U/L
2^*^	Creatinine	168.6 μmol/L	76.8–146.7 μmol/L
4	Albumin	35.8 g/L	27.4–35.7 g/L
5	Albumin	35.8 g/L	27.4–35.7 g/L
12^*^	Lactate dehydrogenase	826 U/L	<640 U/L
13	Bilirubin, direct	7.5 μmol/L	3.3–7.4 μmol/L
17	Urea	7.4 mmol/L	3.0–7.1 mmol/L
18^*^	Eosinophils Alkaline phosphatase	0.82 x 10^9^ cells/L 318 U/L	<0.7 x 10^9^ cells/L <260 U/L
19^*^	Mean corpuscular hemoglobin Mean corpuscular hemoglobin concentration Lactate dehydrogenase	1.25 fmol 24.03 mmol/L 780 U/L	0.9–1.2 fmol 20.8–23.5 mmol/L <640 U/L

The table shows all parameters that were above the reference ranges given in Bauer and Keresztes (23). The donors with mild to moderate changes that were grouped as abnormal are marked with an asterisk.

### 3.2. Impact of matrix effects

Putative matrix effects were observed for IFN-γ, TNFα, IL-4, IL-6 and, to a lesser extent, IL-10. For IL-1β, matrix effects appeared to be only marginal. In IFN-γ, TNF-α-, IL-4- and IL-6-spiked serum, the measured absorbance was reduced to <50% of the absorbance of the corresponding spiked reagent diluent assay buffer sample. In IL-10-spiked serum, the absorption was reduced to approximately 65%. Diluting the serum decreased this effect, but it was still evident in serum diluted 1:10 (Supplementary Figure 1).

### 3.3. Cytokines in different blood products

**Figure 2:**
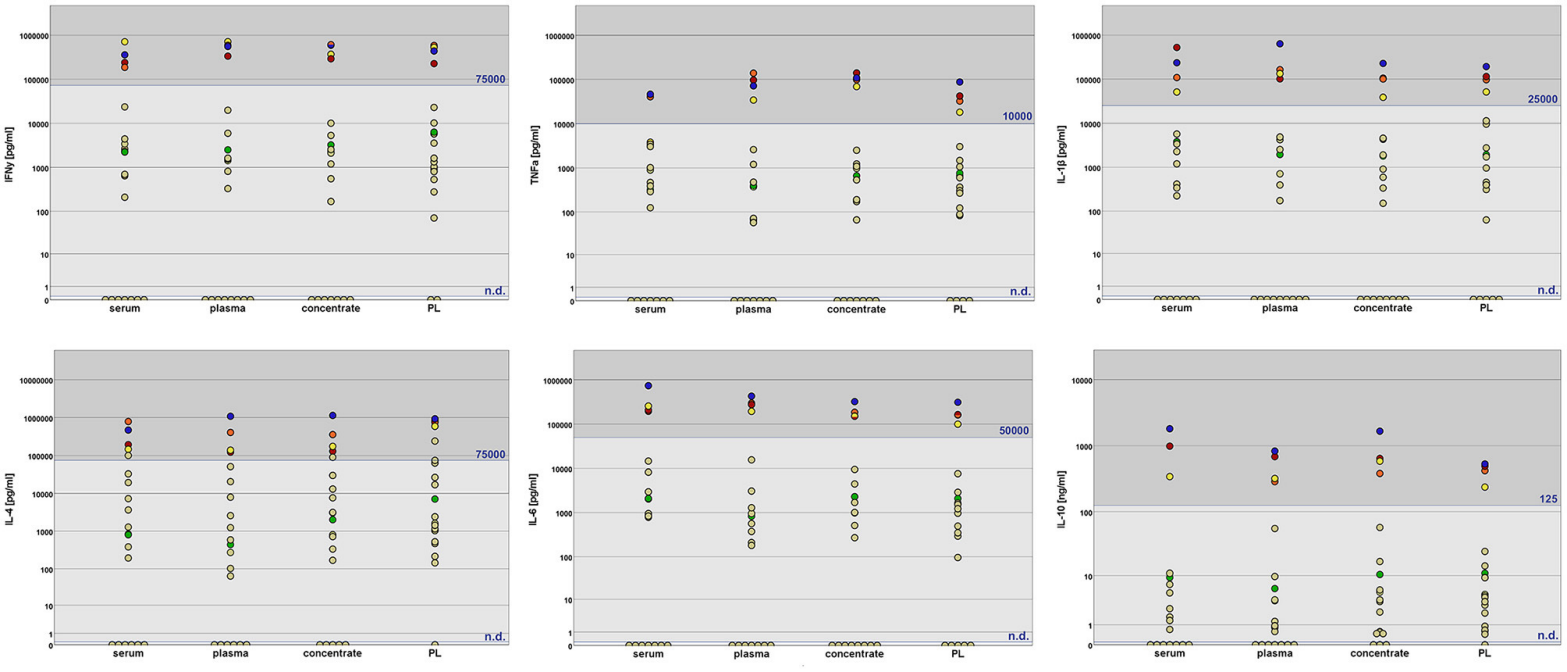
Cytokine concentrations in equine serum, plasma, platelet concentrate and platelet lysate (PL) as measured by sandwich ELISA. The assigned concentration categories are indicated by dark gray (category 3), gray (category 2) and light gray (category 1) background. The thresholds between category 2 and 3 are given in blue letters; category 1 corresponds to no detectable cytokine content (n.d.). Data from donors with abnormal findings in blood chemistry are highlighted in different colors (horse 1: orange; horse 2: red; horse 12: green; horse 18: yellow; horse 19: blue). The cytokine concentration categories were significantly higher in these animals as compared to the others (*p* < 0.01, except for IL-4 in PL).

The concentrations of cytokines in the blood products were highly variable between individual horses. For each cytokine, there were a number of blood donors with no measurable concentration (category 1), donors with medium concentrations (category 2), as well as donors with very high concentrations (category 3). Yet interestingly, the number of donors with no detectable cytokine content was reduced during blood processing to PL, suggesting some cytokine release during freeze-thawing of the concentrates in these samples (Figure 2). Nevertheless, considering all donors, cytokine concentrations remained similar between the different blood products, with no significant differences in concentration categories in the *post-hoc* tests. Instead, the cytokine concentration categories correlated between the different blood products. These correlations were very strong between serum, plasma and concentrate, and more moderate when these were compared with PL (Table 2).

**Table 2:** Correlations of cytokine concentration categories between the different blood products.

	**Plasma**	**Concentrate**	**PL**
**IFN-**γ			
Serum	*p* < 0.001 *r* = 0.948	*p* = 0 *r* = 1.000	*p* < 0.001 *r* = 0.789
Plasma		*p* < 0.001 *r* = 0.948	*p* < 0.001 *r* = 0.770
Concentrate			*p* < 0.001 *r* = 0.789
**TNF-**α			
Serum	*p* < 0.001 *r* = 0.965	*p* < 0.001 *r* = 0.965	*p* < 0.001 *r* = 0.770
Plasma		*p* = 0 *r* = 1.000	*p* < 0.001 *r* = 0.857
Concentrate			*p* < 0.001 *r* = 0.857
**IL-1**β			
Serum	*p* < 0.001 *r* = 0.902	*p* < 0.001 *r* = 0.948	*p* < 0.001 *r* = 0.784
Plasma		*p* < 0.001 *r* = 0.948	*p* < 0.001 *r* = 0.828
Concentrate			*p* < 0.001 *r* = 0.861
**IL-4**			
Serum	*p* < 0.001 *r* = 0.960	*p* < 0.001 *r* = 0.954	*p* = 0.005 *r* = 0.600
Plasma		*p* < 0.001 *r* = 0.909	*p* = 0.026 *r* = 0.498
Concentrate			*p* = 0.004 *r* = 0.616
**IL-6**			
Serum	*p* = 0 *r* = 1.000	*p* < 0.001 *r* = 0.948	*p* = 0.011 *r* = 0.554
Plasma		*p* < 0.001 *r* = 0.948	*p* = 0.011 *r* = 0.554
Concentrate			*p* = 0.003 *r* = 0.631
**IL-10**			
Serum	*p* = 0 *r* = 1.000	*p* < 0.001 *r* = 0.784	*p* < 0.001 *r* = 0.710
Plasma		*p* < 0.001 *r* = 0.822	*p* < 0.001 *r* = 0.764
Concentrate			*p* < 0.001 *r* = 0.877

Furthermore, the concentration categories of the different cytokines compared with each other correlated significantly, revealing that specific donors had either low, medium or high overall cytokine levels, widely irrespective of the type of cytokine. Again, these correlations were very strong in serum, plasma and concentrate, but more moderate in PL (Table 3).

**Table 3:** Correlations between the different cytokines in the respective blood products.

	**IL-1β**	**IL-4**	**IL-6**	**IL-10**	**TNF-α**
**Serum**					
IFN-γ	*p* = 0 *r* = 1.000	*p* < 0.001 *r* = 0.917	*p* = 0 *r* = 1.000	*p* = 0 *r* = 1.000	*p* < 0.001 *r* = 0.965
IL-1β		*p* < 0.001 *r* = 0.917	*p* = 0 *r* = 1.000	*p* = 0 *r* = 1.000	*p* < 0.001 *r* = 0.965
IL-4			*p* < 0.001 *r* = 0.917	*p* < 0.001 *r* = 0.908	*p* < 0.001 *r* = 0.873
IL-6				*p* = 0 *r* = 1.000	*p* < 0.001 *r* = 0.965
IL-10					*p* < 0.001 *r* = 0.965
**Plasma**					
IFN-γ	*p* < 0.001 *r* = 0.948	*p* < 0.001 *r* = 0.901	*p* < 0.001 *r* = 0.948	*p* < 0.001 *r* = 0.948	*p* < 0.001 *r* = 0.948
IL-1β		*p* < 0.001 *r* = 0.862	*p* < 0.001 *r* = 0.902	*p* < 0.001 *r* = 0.902	*p* < 0.001 *r* = 0.902
IL-4			*p* < 0.001 *r* = 0.947	*p* < 0.001 *r* = 0.947	*p* < 0.001 *r* = 0.947
IL-6				*p* = 0 *r* = 1.000	*p* = 0 *r* = 1.000
IL-10					*p* = 0 *r* = 1.000
**Concentrate**					
IFN-γ	*p* < 0.001 *r* = 0.948	*p* < 0.001 *r* = 0.873	*p* < 0.001 *r* = 0.948	*p* < 0.001 *r* = 0.822	*p* = 0 *r* = 1.000
IL-1β		*p* < 0.001 *r* = 0.843	*p* < 0.001 *r* = 0.889	*p* < 0.001 *r* = 0.798	*p* < 0.001 *r* = 0.948
IL-4			*p* < 0.001 *r* = 0.843	*p* = 0.006 *r* = 0.609	*p* < 0.001 *r* = 0.873
IL-6				*p* < 0.001 *r* = 0.798	*p* < 0.001 *r* = 0.948
IL-10					*p* < 0.001 *r* = 0.822
**PL**					
IFN-γ	*p* < 0.001 *r* = 0.845	*p* = 0.010 *r* = 0.559	*p* < 0.001 *r* = 0.845	*p* < 0.001 *r* = 0.928	*p* < 0.001 *r* = 0.885
IL-1β		*p* = 0.023 *r* = 0.505	*p* = 0.002 *r* = 0.644	*p* < 0.001 *r* = 0.804	*p* < 0.001 *r* = 0.822
IL-4			*p* = 0.002 *r* = 0.646	*p* = 0.006 *r* = 0.594	*p* = 0.020 *r* = 0.517
IL-6				*p* < 0.001 *r* = 0.804	*p* < 0.001 *r* = 0.700
IL-10					*p* < 0.001 *r* = 0.834

### 3.4. Cytokine levels and donor health status

To find reasons and predictors for the high inter-individual variability in the blood product cytokine concentrations, we searched for links between cytokine contents and health status. Clearly higher cytokine concentrations (category 3 for most cytokines) had been detected in horses 1, 2, 18 and 19 as compared to the other horses. Interestingly, these were 4 out of the 5 horses that had shown elevated readings in the blood chemistry parameters, namely alkaline phosphatase, creatinine and lactate dehydrogenase (Table 1). The remaining horse (horse 12) with an abnormal blood chemistry finding, however, had middle range cytokine concentrations in its blood products (category 2). Nevertheless, cytokine concentration categories were significantly higher in the donor group with abnormal blood work results than in the donor group with physiological blood work results (*p* < 0.01 for all cytokines and blood products analyzed, except for IL-4 in PL).

## 4. Discussion

The primary aim of this study was to gain insight into the cytokine content of PL and related blood products. Anticipating the high inter-individual differences, we also aimed to identify putative predictors for cytokine content.

The high inter-individual differences in cytokine concentrations between the horses were the most conspicuous finding of the current study, which illustrated that it will be difficult to establish robust reference ranges for serum analysis or quality control thresholds for platelet concentrate and PL products. For serum, such variations are described in the literature, especially regarding IFN-γ concentrations due to vaccination or viral antigen exposure (24). In the current study, however, at the time of blood sampling, none of the horses had received any vaccinations or other medications in the last 2 weeks, and all had received standard vaccinations over the past years.

This study demonstrated that there is a relationship between high cytokine concentrations in blood products, including PL, and increased values in blood chemistry parameters (lactate dehydrogenase, alkaline phosphatase and creatinine). In accordance with this, previous studies showed that there are correlations for the cytokines IFN-γ and TNF-α with the levels of alkaline phosphatase and lactate dehydrogenase (25–27). Probably, the increase in cytokine concentration can be explained by inflammatory processes in the organs such as liver, kidney, muscle or bone, for which these enzymes are considered as indicators.

Cytokines serve as messengers in the regulation of immune response and inflammation. Traditionally, they are divided into pro- (IFN-γ, IL-1β, IL-6 and TNF-α) and anti-inflammatory cytokines (IL-4 and−10), which interact with each other (28, 29). Cytokines are mainly produced by TH1 and TH2 lymphocytes, natural killer as well as mast cells and macrophages, as a result of stimulation by antigens or signaling molecules (24). Due to their contribution to pathological processes, they are being explored as biomarkers for various diseases (30). The exact relationship of cytokine levels and blood chemistry changes in horses will need to be further explored. However, the findings of this study suggest the possibility that blood chemistry measurement might be a convenient tool to estimate the level of cytokines in donor blood and thus facilitate the targeted use of PL.

So far, there are two different operational areas for PL. One is the direct local therapeutic application, e.g., for treatment of osteoarthritis in humans (31, 32) and horses (33), for tendon lesions in humans (34), to promote healing of corneal ulcers (35–37) or for wound healing (38, 39). All beforementioned publications describe an improved outcome compared to control populations. The decisive factors for clinical efficacy are presumably the growth factors contained in the PL, as well as anti-inflammatory cytokines, which can locally influence the inflammatory process (40). On the other hand, PL is already successfully used as a medium additive for cell culture of MSC, which can promote the cellular potency (6, 19, 41, 42). While this has been largely attributed to the growth factors contained in the PL, e.g., VEGF (19), other mediators should be considered as well. As already described by Barrachina et al. (43), stimulation with IFN-y or TNF-α in culture improves the immunomodulatory potency of MSC. Therefore, local application of primed MSC can be expected to yield better therapeutic results. The appropriate amount of pro-inflammatory cytokines is important, because an excess of them impairs the differentiation potential and proliferative capacity of MSC (44). At this point, cytokines contained in the PL may also play a crucial role. With specific levels of pro-inflammatory cytokines, PL alone could possibly serve as a medium additive for priming.

In this study, cytokine levels were measured by sandwich ELISA in different blood products, such as serum, plasma, concentrate and PL. It became apparent that this method has its shortcomings regarding the exact quantification of low cytokine concentrations, as matrix effects occurred in samples measured at low dilutions. We aimed to overcome this on the one hand by repeated measurements at different dilutions and using the highest possible dilution for further analysis, and on the other hand by grouping the cytokine concentrations into categories, the latter to circumvent statistical analysis with inaccurate numbers. Transforming the continuous results data into ordinal data, however, limited the opportunities of inductive statistical analysis. To account for possible confounding variables and interactions of different factors, a generalized linear mixed model would have been more suitable than the analysis approach presented. However, only a multinomial model considering sample type and health status was applicable based on the given data set. This model confirmed that there was no interaction between sample type and health status, but it strongly misestimated the probabilities for the different cytokine categories in different groups, due to their uneven distribution between groups and the relatively small sample sizes per group. Therefore, although this model overall revealed the same trends, namely a significant influence of the health status but no major influence of the sample type on cytokine concentrations, we choose to present a data analysis based on basic group comparisons. While the shortcomings of the ELISA assay and the resulting statistical simplification represent limitations of the current study, it is important to note that the measurements of high cytokine concentrations can still be considered as reliable, as these could be performed with high dilution factors, preventing matrix effects.

In conclusion, this study illustrates that blood products, including PL, are subject to wide variations in cytokine content, which makes careful consideration regarding their use important. It is possible that blood chemistry parameters may provide clues to cytokine content in blood products in individual horses. If this can be confirmed and specified in future research, it would provide a prerequisite to targeted use of PL in clinic and laboratory.

## Data availability statement

The original contributions presented in the study are included in the article/Supplementary material, further inquiries can be directed to the corresponding author.

## Ethics statement

The animal study was reviewed and approved by Regierungspräsidium Gießen. Written informed consent was obtained from the owners for the participation of their animals in this study.

## Author contributions

JM: conception of the study and complete experimental design (together with JB), blood collection, sample and data analysis, data interpretation, and drafting of the manuscript. AH: substantial contribution to the experimental design, blood collection, and processing and analysis. SN: substantial contribution to the experimental design and data interpretation. KB: data analysis and interpretation (together with JB and JM). JB: conception of the study and complete experimental design (together with JM), data interpretation, and drafting of the manuscript (together with JM). All authors have critically revised the manuscript for important intellectual content and approved the publication of its content.
