# Supplementary material for: Cytokines in equine platelet lysate and related blood products
Source: Front Vet Sci. 2023 Mar 9;10:1117829. doi: 10.3389/fvets.2023.1117829 (PMC10033973; doi:10.3389/fvets.2023.1117829)
Supplement: Supplementary file 1 [file Data_Sheet_1.PDF]

frontiers

Supplementary Material

Cytokines in equine platelet lysate and related blood products

Julia Moellerberndt, Alina Hagen, Sabine Niebert, Janina Burk*

*** Correspondence:** janina.burk@vetmed.uni-giessen.de

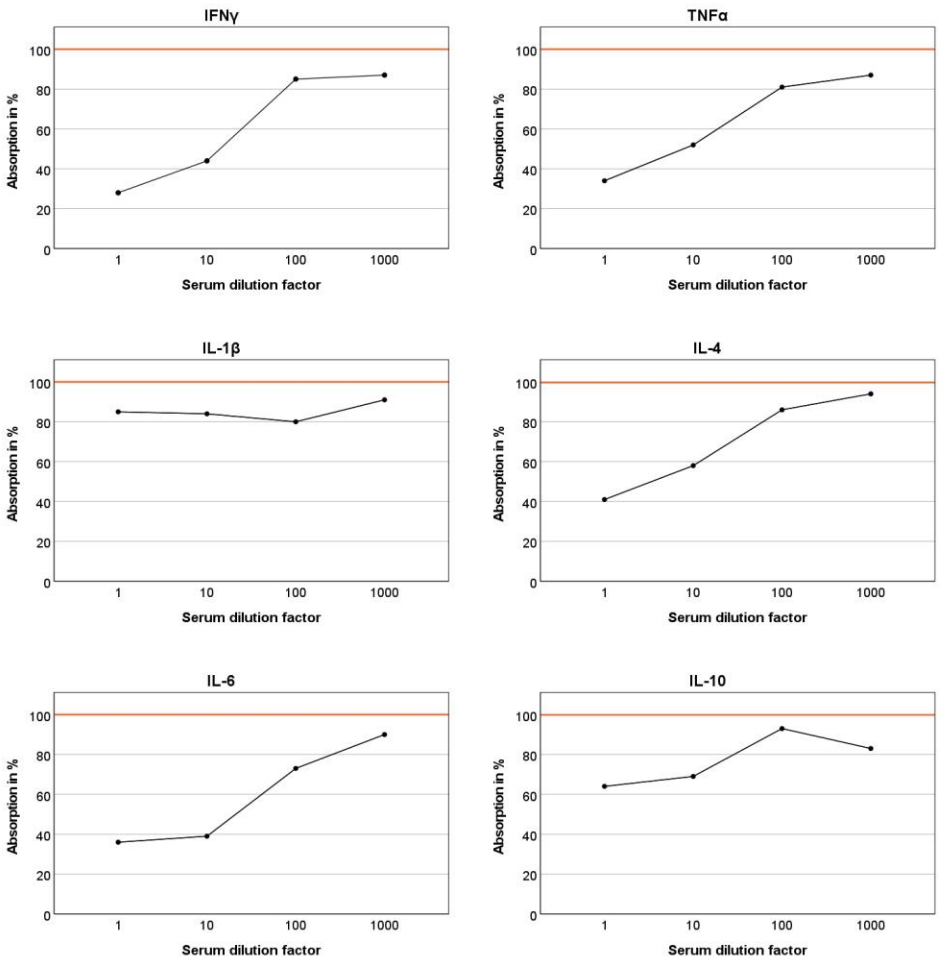

Supplementary Figure 1. Absorption measured in serum samples in different dilutions spiked with cytokine standard, shown as percentage of the absorption measured in correspondingly spiked RD buffer. To estimate the possible influence of serum matrix effects in different serum dilutions, spiking experiments were performed. For this purpose, serum from horses with no detectable cytokine content was pooled and a dilution series (1:1, 1:10, 1:100, 1:1,000 in RD buffer) was prepared. These diluted serum samples were then spiked with cytokine standard, corresponding to the second highest concentration of the assay standard curve for each respective cytokine. The spiked samples were measured using Equine DuoSet ELISA kits (R&D Systems, Minneapolis, MN, USA) according to the manufacturer's instructions. The figure illustrates that for most cytokines, serum matrix effects reduced the absorption by more than 20% when serum was not diluted or diluted 1:10.
